# Assessment of Organ Quality in Kidney Transplantation by Molecular Analysis and Why It May Not Have Been Achieved, Yet

**DOI:** 10.3389/fimmu.2020.00833

**Published:** 2020-05-12

**Authors:** Seraina von Moos, Enver Akalin, Valeria Mas, Thomas F. Mueller

**Affiliations:** ^1^Division of Nephrology, University Hospital Zürich, Zurich, Switzerland; ^2^Division of Transplantation Surgery, Montefiore Medical Center, New York City, NY, United States; ^3^Division Transplantation Surgery, University of Tennessee Health Science Center, Memphis, TN, United States

**Keywords:** marginal organs, molecular diagnostics, implant biopsies, organ quality, surrogate marker

## Abstract

Donor organ shortage, growing waiting lists and substantial organ discard rates are key problems in transplantation. The critical importance of organ quality in determining long-term function is becoming increasingly clear. However, organ quality is difficult to predict. The lack of good measures of organ quality is a serious challenge in terms of acceptance and allocation of an organ. The underlying review summarizes currently available methods used to assess donor organ quality such as histopathology, clinical scores and machine perfusion characteristics with special focus on molecular analyses of kidney quality. The majority of studies testing molecular markers of organ quality focused on identifying organs at risk for delayed graft function, yet without prediction of long-term graft outcome. Recently, interest has emerged in looking for molecular markers associated with biological age to predict organ quality. However, molecular gene sets have not entered the clinical routine or impacted discard rates so far. The current review critically discusses the potential reasons why clinically applicable molecular quality assessment using early kidney biopsies might not have been achieved yet. Besides a critical analysis of the inherent limitations of surrogate markers used for organ quality, i.e., delayed graft function, the intrinsic methodological limitations of studies assessing organ quality will be discussed. These comprise the multitude of unpredictable hits as well as lack of markers of nephron mass, functional reserve and regenerative capacity.

## Background

Good *organ quality* is the basis for successful long-term transplant outcome. The ability to withstand and repair immune and non-immune mediated injury and the number of nephrons to match the increased and persistent metabolic demand to a single kidney characterize optimal kidney organ quality with the potential to best long-term function. Hence, a robust assessment of kidney quality at time of transplantation is needed, in particular in donors with suboptimal conditions, i.e., marginal donors with old age, uncertain medical history, long ischemia time or pre-donation renal failure. In case of doubt clinicians will err on the side of caution and decide on discarding the organ, despite organ shortage and growing waiting lists. This is reflected in the high kidney discard rates in the US despite significant efforts to expand the donor pool. Nearly 20% of kidneys recovered are discarded, mainly based on procurement biopsies as method to assess organ quality (1–4). In Europe, where procurement biopsies are rarely performed, kidney discard rates are significantly lower and this is associated with saved patient life years (4, 5). This difference between US and European allocation practice underscores the need for more reliable and objective methods for organ quality assessment, especially in marginal donors, to decrease the number of discarded organs. So far, no evaluation process has sufficient discriminatory potential to guide the clinician and implanting surgeon team whether to accept or discard an organ. Currently available methods for assessment of organ quality are summarized in Table 1 and discussed in the following paragraphs.

**TABLE 1:** Comparison of different assessment tools to evaluate organ quality in kidney transplantation.

Method		Scores	Strength	General intrinsic limitations
Organ inspection by surgeon			Identification of renal tumors and vascular and anatomical variations and quality of perfusion after retrieval	Interobserver variability, unclear predictive value

Kidney biopsy		Different scores evaluating either individual lesions or composite scores e.g., Banff Score Pirani-Remuzzi Score Maryland Aggregate Pathology Index (MAPI)	Offers the potential to detect preexisting lesions associated with donor medical diseases, e.g., hypertension, diabetes mellitus	Interobserver variability Interpretation on frozen sections differ from paraffin embedded formalin fixed samples. This however is time consuming (up to 5 hours), hence increasing cold ischemia time Sampling error (wedge versus needle biopsy different results) Low predictive value on outcome

Clinical classification models		Classification in SCD/ECD (introduced in 2002)	Practical for clinical routine application (easy, quick, information available at time of decision making)	Categorical classification underestimating variability Original model defining ECD did not include validation cohort
	
		Donor risk scores Kidney Donor Risk Index (KDRI) (including 14 variables) Donor-only KDRI (including 10 donor characteristics) (most recently introduced)	Assessment of graft quality as a spectrum Can easily be calculated based on donor factors	Does not account for injury during procurement Does not account for anatomical abnormalities Does not account for any recipient parameter (including immunological risks) Overall c statistic low with 0.62; c statistic for upper and lower quartile of KDRI 0.78 Falsely elevated in HCV + organs and increased creatinine due to acute kidney injury Not intended to be used as discriminatory tool to determine discard/acceptance but to better characterize organs

Machine perfusion characteristics	Hypothermic machine	Renovascular resistance index		Overall c statistic low with 0.58 for prediction of DGF, association with graft survival unclear
	perfusion	Biomarkers within perfusate		Biomarkers (e.g., NAG, H-FABP, miR21), predictors of DGF but not graft survival
	
	Normothermic machine perfusion	Assessment of functional parameters		EVKP score (macroscopic appearance, blood flow, urine output), urine biomarkers (Endothelin 1, NGAL, KIM 1 and others)

## Currently Available Methods for Assessment of Organ Quality

### Histopathology

In 1995, the seminal paper on procurement biopsies by Gaber et al. presented a significantly increased rate of delayed graft function (DGF) and graft loss with glomerulosclerosis of >20% (6). However, accumulating data in the last 25 years questions the utility of procurement biopsies for evaluating donor kidneys (3, 7, 8). A systematic review by Wang et al. reported that all 47 published studies on kidney biopsies were retrospective, poor in design, and the results were heterogeneous. The percent glomerulosclerosis was most often examined and failed to predict graft failure in 7 out of 14 studies (7).

Analyzing biopsy findings it is necessary to distinguish between pre-implantation biopsies, performed immediately before implantation, and procurement biopsies, taken at time of donor kidney retrieval (9). Histology, in contrast to molecular changes, is expected to be similar in pre-implantation and procurement biopsies. For allocation purposes, focus lies on the procurement biopsy. As time is an important factor in the allocation process, these biopsies are evaluated on frozen sections stained with hematoxylin-eosin and not in paraffin-embedded tissues stained with periodic-acid-schiff, masson trichrome, and methenamine silver. Also, evaluation is done by on-call pathologists often not by an experienced renal pathologist. Furthermore, no consensus exists regarding use of wedge biopsies or core needle biopsies. All these factors pose problems. Hence, classification of histological lesions might differ when evaluated on frozen *vs.* paraffin embedded sections and interpretation might vary between on-call pathologists vs. experienced nephro pathologists contributing to the poor quality with missing information, lack of concordance and reproducibility (8–12). Even the agreement between expert renal transplant pathologists were only moderate to poor at Banff Histopathological Consensus meeting for preimplantation kidney biopsies with most interclass correlations less than 0.5 (12). In addition, intrinsic differences between wedge biopsies, that preferentially evaluate the subcapsular zone overestimating glomerulosclerosis, and core needle biopsies, that preferentially represent the cortex, further impact comparisons between various practices of procurement biopsies (9).

Finally, no consensus exists regarding the grading system to be used for interpretation of procurement biopsies. Besides the Banff grading system scoring individual lesions (12), several composite histological scoring systems have been described (7, 9). Yet, most histological composite scores lack validation in independent cohorts as well as testing of their predictive power in multivariate analyses including donor age and organ function and hence they might erroneously appear as independent predictors of graft failure. These facts underline the difficulty to predict long-term graft outcome based on histological evaluation of procurement biopsies (13). All these limitations translate into high discordance between two biopsies obtained of the same kidney (8) and also contribute to the high discrepancy in discard rates between centers (2, 3).

Graft survival rates of unilaterally discarded kidneys might indeed still be acceptable for some patients (2, 8). One-year death censored graft survival rate of recipients from unilaterally discarded kidneys due to donor factors (in particular biopsy findings) has been reported to be over 90% and five-year death censored graft survival was >85% (2). This underscores the fact that the currently available scores for organ assessment using histology inaccurately capture organ quality and the gain of life years for the individual patient.

### Clinical Scores

The first clinical parameter found to be negatively associated with graft survival was age (13, 14). Besides age, established cardiovascular co-morbidities also associate with graft survival (15). Hence, common variables used in all scoring systems include donor age, history of hypertension and serum creatinine, altogether being surrogate markers of reduced nephron mass and extent of established injury and repair capacity, key donor factors contributing to long-term graft outcome (13, 16). However, these clinical markers lack robustness and standardization as organ quality metrics.

The recently introduced KDRI score (resp. kidney donor percentile index KDPI) reflects the rate of graft failure relative to a healthy 40-year old donor. This score was originally based on 14 donor characteristics (donor age, race, history of hypertension, history of diabetes, serum creatinine, cerebrovascular cause of death, height, weight, donation after cardiac death, hepatitis C virus status, HLA-B and -DR mismatch, cold ischemia time, *en-bloc* kidney transplant, dual kidney transplant) (17–19) and later reduced to 10 variables, as some information may be missing at time of transplantation. This is a far more granular tool for physicians to evaluate the offer and assess generic donor quality and outcome than the previously used dichotomous extended criteria donor (ECD) vs. non-ECD classification. Yet, despite introduction of this more detailed risk index, discard rates in the US remained unchanged at roughly 18–20% (20).

The differences in discard policies and application of the KDRI scores are highlighted in a recently published analysis by Aubert et al. (4). The probability of organ discard for the same KDRI is significantly higher in the United States compared to France and the interpolation of a similar organ use strategy in the United States would generate additional 132’445 allograft life-years over a ten-year observation period with greatest gain of life years through reduced discard of the organs with highest KDRI. These differences in applying the KDRI for accepting organ offers also reflect its limited predictive power. A recent study showed no significant difference in 5-year death-censored graft survival between DCD KDPI 61–81 and DCD KDPI ≥ 85 when used for donation after cardiac death (DCD) kidneys (18). In line with the limited discriminative power regarding graft failure very high KDPI kidneys may reveal acceptable outcomes (21–24). Another group showed 5-year graft survival of 91% using kidneys with KDPI score of 97% as dual transplants, highlighting that besides KDRI, nephron mass plays a major role with respect to graft survival (14, 25). A further a critical issue when using KDRI/KDPI is Hepatitis C virus (HCV) status having the largest contribution to KDPI (KDRI b coefficient 1/4.24; “Xb” component 1/4.24). However, HCV + kidneys are mostly young donors and at current era with available excellent antiviral treatment for HCV, clinical outcomes are excellent in HCV negative patients receiving HCV + deceased-donors (26). The other important critical component of KDPI is the pre-donation serum creatinine level, which might be “falsely” high due to acute kidney injury from acute tubular necrosis. In a multicenter deceased donor study of 2,430 kidneys transplanted from 1,298 deceased donors 585 (24%) were from donors with AKI. The analysis did not show any significant difference in graft survival at 4 years by donor AKI stage (27).

All these articles question the utility of KDRI/KDPI as single decision tool with respect to kidney discard policies. Even though KDRI/KDPI has repeatedly been shown to associate with graft failure, a high KDRI/KDPI is not synonymous with graft failure and underscores its limited discriminative power as single decision tool.

### Machine Perfusion

Research and applications of machine-based organ preservation have experienced a significant revival with the goals to reduce peri-transplant ischemia reperfusion injury, to facilitate assessment of organ quality and directed organ therapies, and to decrease the number of marginal organs to be discarded. First described as early as 1935 by Carrel and Lindbergh (28), interest in organ perfusion has re-emerged with the landmark trial published by Moers et al. (29). Machine perfusion for organ preservation was associated with a reduction of DGF compared to cold storage and its application has led to reduced discard rates of organs (30). However, these positive effects on short-term function did so far not translate into a marked improvement in long-term outcomes (31, 32). However, more sophisticated perfusion methods and cell-based therapies are investigated. Currently hypothermic machine perfusion is the most widely used technique, in recent years normothermic machine perfusion is gaining interest (33).

In addition to positively impacting reperfusion injury and organ preservation, machine perfusion also offers the opportunity for organ quality assessment based on perfusate analysis or measurement of perfusion dynamics such as intravascular renal resistance. The largest randomized controlled trial prospectively assessing renal intravascular resistive indexes on hypothermic machine perfusion and its association with graft outcome by Jochmans et al. showed that renal resistance at the end of hypothermic machine perfusion is an independent risk factor for both DGF and 1-year graft failure, yet the predictive power was low with a c-statistic of only 0.58 (34). Similar findings are reported by de Vries et al. and Parikh et al., showing only modest correlation with early graft function (35, 36). Likewise, perfusate analyses indicated that biomarkers, such as NAG or H-FABP, are associated with DGF, but again with low predictive value in differentiating functioning versus non-functioning grafts (37). Another group described levels of microRNA-21 (miR-21) to correlate with early graft function, but no data on association with long-term graft function is available (38). Hence, so far, neither dynamic machine perfusion characteristics such as renal resistance nor machine perfusate biomarkers can be used as stand-alone criteria for organ quality assessment with sufficient precision (36, 39).

Yet, novel techniques using normothermic perfusion allow for further assessments of functional parameter in addition to the above described flow/resistance markers. Hosgood et al. (40). described an *ex vivo* kidney perfusion quality assessment score (EVKP score) based on macroscopic appearance, renal blood flow and urine output after *ex vivo* normothermic kidney perfusion correlating with DGF but not long-term outcome. The same group correlated urine biomarkers of injury with this score. They measured a significant correlation between levels of urinary endothelin-1 and NGAL and perfusate parameters as well as between the EVKP score and donor creatinine at organ retrieval, while no correlation was found for KIM-1 (41). Similar results, reporting a lack of correlation of KIM-1 with donor AKI, have also been reported by other groups, likely due to the fact that KIM-1, in contrast to NGAL, is a rather late marker of kidney injury. However, the predictive power of these urinary biomarkers, despite being sensitive for structural kidney damage, is still unclear. Of note, a large multicenter deceased donor study of 2,430 kidney transplant recipients from 1298 donors did not find an association of the donor urine injury biomarkers microalbumin, NGAL, KIM-1, IL-18, and L-FABP with graft failure at a median follow-up of 4 years, questioning the predictive utility of urinary biomarker measurements during normothermic *ex vivo* perfusion (42).

Future evaluations will show whether novel techniques, such as normothermic machine perfusion, may allow better assessment of organ quality and function under near-physiological conditions (43). A key advantage of machine perfusion might well be the additional time gained for organ evaluation and the clinical decision to use or not use the organ.

## Molecular Diagnostics Using Kidney Implantation Biopsies

As outlined above, evaluation of organ quality by clinical scores, histopathology or perfusion characteristics lacks discriminatory power to guide clinicians to accept or discard an organ, in particular in the situation of marginal donors.

Over the recent years molecular analysis of biopsy samples has become a reliable, technically robust, not too expensive methodology including transcriptome, proteome and metabolome technologies. The unbiased, quantitative “omics’ approaches have become standard of care in oncology, classifying tumors and individualizing therapy. Hence, great expectations have been based on molecular diagnostics as they potentially offer an alternative, more objective and quantitative method for organ evaluation. Molecular profiling indeed demonstrated to go beyond histopathologic evaluation being able to detect changes not captured by histopathology. In a previous review we have summarized molecular studies of 0-hour biopsies (both pre-implantation and post-reperfusion) published till 2010 (44). It could be shown that transcriptome profiles provide a quantitative measurement of inflammatory burden, detect coordinated activation of pathways of immune activation, defense response, oxidative stress and a parallel inhibition of metabolism and transport or ion binding. In particular, transcriptome patterns identified changes in kidneys such as susceptibility to DGF, which was not reflected using clinical and histopathological scores (45). However, despite the number of promising findings, no robust set of predictive molecular markers for organ quality measurement had been identified in these early studies.

Since then a number of new studies have been conducted to further assess the potential to evaluate organ quality and transplant outcomes. Table 2 summarizes studies on molecular analyses of peri-transplant biopsies assessing organ quality that have been published since 2011 and are listed in PubMed.

**TABLE 2:** Molecular diagnostics of early kidney transplant biopsies [summarizing studies published since 2011, studies published before were previously summarized (44)].

References	Time of biopsy	Patient/biopsy numbers	Follow up	Test and outcome markers	Findings	Strengths and limitations with focus on quality assessment

*DGF as surrogate for organ quality and early outcome*						
(46)	Implantation^1^	34 DD + 9 controls (tumor-nephrectomies)	Early post-TPL	4 candidate AKI genes studied in micro-dissected tubulointerstitial vs. glomerular segments: KIM1 (i.e., HAVCR1), NGAL (i.e., LCN2), CYR61, NTN1 *Tested outcome: DGF*	• Upregulation of NGAL and KIM1 in DGF • In multivariate model only D age significantly associated with DGF	**Strengths:** • Analysis of micro-dissected samples **Limitations:** • No validation set • Low numbers • No long-term outcome

(47)	6 weeks post-TPL	107 in total: 14 LD + 93 DD Indication biopsies	≥ 2 years	Pathogenesis based transcript sets (PBTs) for inflammation and injury *Tested outcome: DGF, GFR*	• The molecular phenotype correlates with previous DGF • No difference in PBTs between LD and DD • Molecular phenotype correlates with 6 month atrophy and scarring • Molecular phenotype did not predict future functional decline or allograft loss	**Strengths:** • long-term analysis incl. assessment of graft loss **Limitations:** • no validation set • No peri-TPL biopsies

(48)	Pre-implantation (back bench)	92 DD (91 analyzed) cold perfusion and pump perfusion	≥ 1 year	Microarrays (validation in independent sample set) *Tested outcome: DGF and 1 month GFR* (≤ / > *45 ml/min/1.73 m^2^)*	• Clinical variables pre-transplant did not identify kidneys with better or poorer function during first year • 1 month function predictive of 1 year function • Low *vs.* high GFR within DGF group differ in inflammation and immune activation transcripts pre-TPL, at month 1 and throughout first year • DGF not associated with 3 month and 1 year function • Molecular phenotype does not separate DGF vs. no DGF	**Strengths:** • validation set • Unbiased gene selection approach • GFR at 1 month and 1 year as outcome marker and not only DGF **Limitations:** • no analysis of longer-term graft function (incl. graft loss)

(53)	Pre-implantation (back bench)	112 biopsies in 100 DD	29 months (median)	Unbiased microarray gene expression approach: four differentially expressed genes selected (validated in an independent set) *Tested outcome: 1 month GFR* (≤ / > *45 ml/min/1.73 m^2^)*	• Groups with high *vs.* low 1 month GFR stay different at 24 months post-TPL • D age only clinical marker different in high *vs.* low 1 month GFR groups, not race, gender, CIT, PRA and cause of death • CCL5, CXCR4 and ITGB2 expression in pre-implantation biopsies discriminate GFR high *vs.* low group at 1 month	**Strengths:** • validation set [overlap with (50)] • Unbiased gene expression approach • GFR up to 24 months as outcome marker **Limitations:** • no analysis of longer-term graft function (incl. graft loss)

(54)	Early post-TPL AKI	28 biopsies (26 patients with AKI)	3.9 years	Unbiased microarray gene expression approach (validation set of 27 kidneys) 11 protocol biopsies *Tested outcome: GFR, graft loss, dialysis*	• No difference in kidney outcome with or without AKI • Histology did not correlate with DGF, GFR, recovery of function or IRRAT score • 30 injury repair response transcripts (IRRATs) in AKI correlate with function, future recovery, need for dialysis but not future graft loss • IRRAT transcripts differ to DGF transcripts	**Strengths:** • validation set • Unbiased gene expression approach • Analysis of AKI in allografts • Analysis of longer-term function, incl. graft loss **Limitations:** • small number • No peri-TPL biopsies

(55)	Implantation^1^	70 biopsies from 53 DD 8 control nephrectomies	4.2 years	AKI associated. transcripts (IRRATs), see Famulski et al. (54) *Tested outcome: early and 1 and 3 year GFR, histology, clinical scores, graft loss*	• D and R age, not histology correlate with early dysfunction • CAVE: age bias, old kidneys are often allocated to old Rs • D age predicts late function • D age, D age-dependent models such as KDRI and Irish and histology correlate weakly with late function • IRRATs predict early but not late function in SCD	**Strengths:** • independently validated gene set • Analysis of clinical, morphological and molecular markers as predictors of early and late function **Limitations:** • no validation set • No longer-term follow-up (incl. graft loss)
(49)	Sequential biopsies: Procurement and Pre-implantation and Implantation^1^	105 biopsies in 38 DD	1 year	92 pre-selected genes associated with I/RI injury *Tested outcome: DGF*	Gene expression heterogeneity increases from procurement to pre-implantation to implantation biopsies suggesting different organ vulnerability • Cold storage not associated with significant transcript changes • Reperfusion associated with activation of innate and adaptive immune response and apoptosis • Low netrin-1 (NTN1) and higher tubular atrophy on histology predictive of DGF	**Strengths:** • investigation of sequential biopsies from the same graft **Limitations:** • no validation set • No long-term follow-up

***Molecular markers for biological age as markers for organ quality***						

(50)	Pre-implantation	120 DD	1 year	Comparison of predictive capacity of biomarkers of aging (CDKN2A expression and telomere length) *Tested outcome: 6 and 12 month function, DGF*	• CDKN2A, stronger than telomere length, predict DGF and 6 and 12 month graft function • Pre-TPL D risk classification based on CDKN2A and ECD criteria possible	**Strengths:** • assessment of markers of biological age (yet additive value to chronological not clear) **Limitations:** • no validation cohort • Short follow-up •No assessment of long-term graft outcome

(51)	Pre-implantation	94 DD	1 year	Pre-selected microRNAs, CDKN2A expression (validation cohort) *Tested outcome: t1/2 creatinine fall, DGF, 3, 6 and 12 month function*	• A score using senescence associated miRNAs (hsa-miR-217, hsa-miR-125b; regulators of CDKN2A) combined with D age and organ type predicts occurrence of DGF (Sens > 90%, Spec > 60%) • CDKN2A expression and hsa-miR-217 correlate positively with 12 month function	**Strengths:** • Investigation of markers of biological age • Concept of allostatic load **Limitations:** • Questionable clear added value of miRNAs and CDKN2A to chronological age alone • No assessment of long-term graft function or loss

(52)	Pre-implantation and Implantation^1^ (paired biopsies)	55 DD	1 year	Unbiased, RNASeq, genome, transcriptome and epigenetics analysis *Tested outcome: DGF, creatinine fall 1*st *week, 3, 6 and 12 month function*	• Transcriptional response to reperfusion injury similar for allografts irrespective of post-TPL outcome, but magnitude is greater for those exhibiting DGF • DGF specific transcripts reveal differential promotor methylation status • Pre-implantation TP53, CDKN1A/p21, CDKN1B/p27 (“BioAge”) associated with 3 and 6 month function • Molecular signature for allostatic load (burden of “wear and tear”) reflects age-related physiological capability and resilience • DGF is a manifestation of its allostatic load	**Strengths:** • Unbiased analysis investigating genome, transcriptome and epigenetics • Concept of allostatic load **Limitations:** • No validation set • Questionable clear added value of biological age markers to chronological age alone • Short term follow-up • No assessment of long-term graft function or loss

***Analysis of cell infiltrates and proteomics***						

(56)	Pre-implantation and 4 months post-TPL	94 biopsies (60 DD and 34 LD)	≤5 years	Pre-selected genes, macrophage infiltration *Tested outcome: graft survival, 4, 24 and 60 month function*	• Baseline expression of selected genes did not correlate with GFR at any time point • Higher pre-implantation levels of inflammation, monocyte recruitment, and M1/M2 macrophage transcripts in DD compared to LD • 4 month fibrosis transcript levels correlate with long-term function in DD • Expression of inflammation and fibrosis markers at 4 months and difference between 4 months and baseline correlate negatively with medium- and long-term renal function in DD • TGFb1 best predictor of long-term renal function in DD	**Strengths:** • Broad gene set • Analysis of graft survival and outcome up to 5 years **Limitations:** • No validation set • LD *vs.* DD impacted by multitude of factors (D/R age, CIT, dialysis time) not *per se* reflecting tissue quality

(57)	Pre-implantation	38 donors (proteomic analysis done in 10 donors) always one mate kidney when both had same 3-months function to select for donor factors	12 months *Tested outcome: 3 and 12 month GFR*	Pilot study, unsupervised proteomics analysis	• Common evaluation methods not predictive of outcome (KDPI, histology, AKI score) • Pathways of cellular response to stress, cell surface receptor signaling and enrichment of reactive oxygen species detoxification differ between optimal and suboptimal donors • Proteomics analysis differentiates between different outcomes	**Strengths:** • Novel “mate kidney approach” analyzing proteomics • Unbiased proteomics analysis **Limitations:** • No validation set • Small number • Short follow-up • Highly selected groups

*^1^Taken post-reperfusion, i.e., within one hour after opening of the anastomosis.*

A majority of studies focused on DGF as a surrogate marker for organ quality and early outcome (46–52). They largely confirm the earlier findings that DGF is usually better predicted with molecular changes than histology or clinical scores at time of transplantation (46, 49–52). They identified molecular changes associated with kidney injury (such as NGAL, syn. LCN2, KIM1, syn. HAVCR1, NTN1) (46, 49) and aging (CDKN2A) (50–52) as DGF biomarkers. However, these peri-transplant changes associated with DGF did not allow a robust prediction of medium- to long-term functional outcomes.

Mas and her group showed that transcript changes associated with early kidney function but not with DGF *per se* correlate with outcome. Kidneys with DGF and also a low GFR at 1-month post-transplant showed inferior medium- to long-term outcomes. The pre-implantation biopsies of these kidneys showed an increased expression of pathways associated with immune activation and inflammation. Gene transcripts of CCL5, CXCR4, and ITGB2 discriminated best between low *vs.* high GFR. This difference in kidney function remained throughout the period of observation of 2 years (48, 53). Findings of the Halloran group confirm the lack of predictive power of gene changes associated with DGF (54, 55). They identified gene transcript changes associated with AKI in transplant biopsies. These so-called injury and repair associated transcripts (IRRATs) correlate with degree of injury, repair capacity and functional outcome but not with DGF (54, 55). However, with a sufficient long-term follow up of more than 2 years, peri-transplant molecular phenotypes at time of, or early after transplantation seem not to correlate with medium- to long-term transplant function. Molecular changes in 6-week protocol biopsies correlated with atrophy and scarring at 6 months but not with future functional decline (47), implant biopsies did not predict late function (54, 55). In contrast, long-term function correlated with histopathology changes associated with aging or clinical scores, in particular donor age (53, 55).

Consequently a number of studies focused on molecular markers for biological age as parameters for organ quality (50–52). In particular increased expression of CDKN2A associated with graft function, probably better reflecting the allostatic load of “wear and tear” of an organ and its resilience to cope with the peri- and post-transplant stressors (50–52). However, the clear added value of markers of biological age like CDKN2A or others like telomere length, microRNAs or epigenetic changes to the simple measurement of chronological age is not clear. In addition, the age allocation bias, i.e., old kidneys are predominantly given to old recipients, and hence likely poorer quality organs are transplanted into recipients with more comorbidities and inferior outcomes, makes it difficult to identify and validate robust quality markers in old kidneys (55).

An interesting, recent study analyzed gene expression in cell infiltrates at time of transplantation and 4 months post-transplant (56). This study indicated gene expression of inflammatory and fibrotic markers at 4 months, and differences between 4 months and baseline, correlated negatively with renal function up to 5 years. Another small, exploratory but cutting-edge methodology study by Kaisar et al. (57). suggests that proteomics analyses are able to discriminate different outcomes that were not predicted by common evaluation methods such as clinical (KDPI), histology or AKI scores (57). These promising studies need further validation and larger numbers.

In general none of the molecular analyses outlined here have entered the clinical routine diagnostics and organ quality is still evaluated exclusively by clinical and histopathology-based scores.

The question is why these molecular analyses have not yet identified robust quality markers and hence successfully translated into clinical useful tests? This might be due to intrinsic limitations of molecular studies, selection of insufficient surrogate markers and end points for outcome studies, or the principal unpredictability of long-term outcomes with donor organ characteristics given heterogeneity and multitude of hits during the post-transplant life of the donor kidney.

Molecular analyses of donor kidney biopsies might not depict structural changes or reflect nephron mass. They measure tissue cell mixtures depending on the location of the biopsy site, cannot predict the multitude of additional immune and non-immune hits and recipient factors that occur in the long run. They are drowned by the tidal wave in expression changes due to brain death and the associated SIRS-like syndrome.

The surrogate markers for kidney quality used for the identification of molecular changes is another likely reason for the lack of established kidney quality profiles. Delayed graft function, chronological rather than biological age, incomplete disease phenotyping, weak markers of kidney function (such as creatinine), short follow-up periods, small samples sizes or lack of validation studies all contribute to the still unfulfilled promise of molecular diagnostics for organ quality assessment.

## Organ Quality Assessment of Non-Kidney Transplants

Pre-transplantation assessments of organ quality in non-kidney solid organs primarily rely on clinical scores and markers assessed during *ex vivo* machine perfusion. Comparable to kidney transplantation there is no established molecular assessment of biopsy samples and few examples are given below. In-depth analysis of organ quality assessment measures for other organs than the kidney is out of the scope of this review.

In liver transplantation, organ quality has been correlated with cumulative bile acid production and coagulation parameters (58). Also metabolomic signatures associated with early graft function comprising key pathways involved in lipid homeostasis and histidine pathway have been described (59). With respect to analysis of molecular markers, investigation of microRNA profiles in graft preservation solutions has been shown to be predictive of ischemic-type biliary lesions after liver transplantation, which are the second most common cause of graft failure after liver transplantation (60). The ratio of hepatocyte to cholangiocyte-derived miRNAs (with special focus of miR 122 and miR 222) was predictive of graft viability (60–62). In pancreas transplantation, assessment of organ quality is performed during machine perfusion measuring insulin secretion, acid-base balance and perfusion characteristics (63). Likewise, in lung transplantation organ quality assessment is reported through ventilation parameters, analysis of arterial blood gases on perfusate samples with recent focus on metabolic components of glucose consumption and lactate production (64). Other groups indicated that levels of inflammatory cytokines (65, 66), endothelin-1 (67), adhesion molecules (68) or neutrophil extracellular traps (69) in lung perfusate are associated with post-transplant primary graft function. Similarly, assessment of donor heart quality prior to transplantation is attempted by analyzing perfusate during machine perfusion (70).

## Critical Discussion of Surrogate Markers Used for Kidney Quality Assessment

The majority of published studies on molecular assessment of organ quality used *DGF*, i.e., transient renal failure immediately post-transplantation, as surrogate marker for graft quality and outcome. This is based on the association of reduced graft survival of DGF kidneys in standard brain death donors (DBD) shown in some, but not in all studies. The limitations of DGF as a surrogate outcome marker for poorer organ quality is highlighted by the excellent quality and long-term outcomes of DCD organs. Despite the high percentage of DGF cases these positively selected cases with usually young age and lack of comorbidities show good long-term outcomes. Similar lack of correlation with longer-term outcomes and DGF is seen analyzing mate kidneys. Donor characteristics rather than ischemia times or DGF rates determine the long-term performance (71–74).

Moreover, definition of DGF is not uniform (71). More than 10 different definitions are used and most importantly none of them was associated with poorer graft survival in DCD kidneys (71). The limitations of DGF as a quality marker is further underlined as so far no treatment of DGF translated into significant improvement in long-term outcome (75).

Patho-physiologically the higher risk of DGF in DCD donors compared to DBD donors can be explained by the unavoidable extended warm ischemia time and associated increased ischemia-reperfusion injury. However, full recovery and excellent long-term graft outcome underline repair capacity and nephron mass as organ quality determinants.

Hence, not DGF *per se* but rather ability to recover from DGF as indicated, e.g., by GFR at 1 month might be a more reliable marker of long-term graft outcome and quality, as recently reported by Lee et al. (76). Donor age, donor final creatinine and cold ischemia time were significantly associated with DGF recovery status (76). DGF is a syndrome and duration of DGF and degree of acute kidney injury is associated with renal outcome, in transplant and non-transplant settings (77, 78). Extent of recovery presumably reflects the intrinsic repair capacity of the donor organ. Age strongly defines repair capacity and this might explain donor age as the most widely used criterion in all clinical scores assessing organ quality pre-transplantation (73, 79).

In summary, the post-transplant course is determined by donor factors, acute peri-transplantation injury as well as recipient factors. DGF *per se* is a poor, but most frequently used, surrogate marker for organ quality (see Table 2). Hence, the focus on identifying DGF-associated molecular patterns might be one reason that so far molecular diagnostics of organ quality has not translated into clinical decision making. In addition, molecular assessment of repair capacity and biological tissue aging is still ill defined. Ongoing work on robust molecular markers of biological age is promising (see Table 2) but again has not yet translated into clinical utility.

Successful organ transplantation is largely defined by a good and long-term functioning kidney graft. This requires a sufficient nephron mass to meet the increased, long-term metabolic demand and stresses of a single kidney in a transplant recipient. In the unstable setting of brain death and organ donation donor serum creatinine or estimated GFR are unreliable markers of nephron mass or reserve capacity. The same applies to histopathology and clinical scores. The identification of molecular markers for nephron mass in addition to repair capacity would be most valuable but yet, has not been achieved. This might be due in part to the lack of long-term studies. As shown in Table 2 most studies focus on short-term function. The identification of molecular changes in peri-transplant biopsies that correlate with long-term function is needed.

## Proposed Approaches to Optimize Molecular Diagnostics for Clinical Routine Application

### Assessment of Nephron Mass by Molecular Methods: Paired Kidney Transplantation Study

• Comparison of molecular profiles at implantation biopsy between kidney pairs from the same donor both with high eGFR at 1 year post transplantation (i.e., eGFR > 60 ml/min/1.73 m^2^) and kidney pairs from the same donor with both low eGFR at 1 year post transplantation (i.e., eGFR < 30 ml/min/1.73 m^2^). This should primarily reflect intrinsic donor factors rather than post-transplant hits and recipient factors. Note: Ratio for taking follow up of 1 year only: if taking too long follow up (longer than 1 year) recipient factors might become additionally relevant.

### Assessment of Kidney Regeneration Capacity: Recovery From AKI (i.e., DGF)

• Comparison of molecular profiles at implantation biopsy between kidney with low delta of expected and observed creatinine at 1 year post transplantation (e.g., delta 25%; i.e., kidney with good regeneration capacity) and kidneys with high delta of expected and observed creatinine at 1 year (delta > 25%; i.e., kidney with impaired regeneration capacity). Note: make sure taking only kidney with good match of recipient/donor weight (i.e., R/D weigh ratio of 0.8–1.2 allowed) (80).

### Assessment of Effect of Pumping on Recovery Form AKI in High Risk Patients (i.e., Patients With Low Regeneration Capacity)

• Once molecular profiles of kidney with low regeneration capacity is characterized: comparison of delta expected-observed creatinine at 1 year in high risk kidneys preserved with pumping as compared to delta expected-observed creatinine in high risk kidneys preserved with cold storage.

## Author Contributions

SM and TM designed the first draft of the manuscript. EA and VM revised the manuscript. All authors approved the final version of the manuscript.

## Conflict of Interest

SM declares a travel grant from the Astellas outside from the submitted work. The remaining authors declare that the research was conducted in the absence of any commercial or financial relationships that could be construed as a potential conflict of interest. The reviewer MN declared a past co-authorship with several of the authors to the handling Editor.

## Abbreviations

AKI: acute kidney injury
CIT: cold ischemia time
D: donor
DBD: donation after brain death
DCD: donation after cardiac death
DD: deceased donor
DGF: delayed graft function
ECD: extended criteria donor
EVKP: score *ex vivo* kidney perfusion score
GFR: glomerular filtration rate
HCV: hepatitis C
I/RI: ischemia reperfusion injury
IRRATs: injury and repair response associated transcripts
KDPI: kidney donor percentile index
KDRI: kidney donor risk index
LD: living donor
MAPI: Maryland aggregate pathology index
PBTs: pathogenesis based transcript sets
PRA: panel reactive antigen
R: recipient
SCD: standard criteria donor
TPL: transplantation.
